# Unifying generative and discriminative learning principles

**DOI:** 10.1186/1471-2105-11-98

**Published:** 2010-02-22

**Authors:** Jens Keilwagen, Jan Grau, Stefan Posch, Marc Strickert, Ivo Grosse

**Affiliations:** 1 Leibniz Institute of Plant Genetics and Crop Plant Research (IPK), Gatersleben, Germany; 2 Institute of Computer Science, Martin Luther University Halle-Wittenberg, Germany

## Abstract

**Background:**

The recognition of functional binding sites in genomic DNA remains one of the fundamental challenges of genome research. During the last decades, a plethora of different and well-adapted models has been developed, but only little attention has been payed to the development of different and similarly well-adapted learning principles. Only recently it was noticed that discriminative learning principles can be superior over generative ones in diverse bioinformatics applications, too.

**Results:**

Here, we propose a generalization of generative and discriminative learning principles containing the maximum likelihood, maximum a posteriori, maximum conditional likelihood, maximum supervised posterior, generative-discriminative trade-off, and penalized generative-discriminative trade-off learning principles as special cases, and we illustrate its efficacy for the recognition of vertebrate transcription factor binding sites.

**Conclusions:**

We find that the proposed learning principle helps to improve the recognition of transcription factor binding sites, enabling better computational approaches for extracting as much information as possible from valuable wet-lab data. We make all implementations available in the open-source library Jstacs so that this learning principle can be easily applied to other classification problems in the field of genome and epigenome analysis.

## Background

Classification of unlabeled data is one of the main tasks in bioinformatics. For DNA sequence analysis, this classification task is synonymous to the computational recognition of short signal sequences in genomic DNA. Examples include the recognition of transcription factor binding sites (TFBSs) [1,2], transcription start sites [3,4], donor or acceptor splice sites [5-7], nucleosome binding sites [8,9], miRNA binding sites [10,11], or binding sites of insulators like CTCF [12].

Many of the employed algorithms use statistical models for representing the distribution of sequences. These models range from simple models like the position weight matrix (PWM) model [1,13,14], the weight array matrix (WAM) model [6,8,15], or Markov models of higher order [16,17] to complex models like Bayesian networks [2,18,19] or Markov random fields [7,20,21]. A wealth of different models has been proposed for different data sets and different biological questions, and it is advisable to carefully choose an appropriate model for each data set and each biological question separately [4,7,22]. However, the performance of a model highly depends on the model parameters learned from training data. In comparison to the effort spent for developing and choosing appropriate models, developing and choosing appropriate learning principles has been neglected, even though this choice is of fundamental importance [23-27] and equally non-trivial.

In the last decades, several learning principles have been proposed for estimating model parameters. The *maximum likelihood *(ML) learning principle [28,29] is one of the first and most popular learning principles used in bioinformatics. An alternative is the *maximum a posteriori *(MAP) learning principle [30] that applies a prior density to the parameters of the models.

The ML and the MAP learning principles are commonly referred to as *generative*. Recently, *discriminative *learning principles have been shown to be promising in several bioinformatics applications [16,17,20,26,27,31]. The discriminative analogue to the ML learning principle is the *maximum conditional likelihood *(MCL) learning principle [24,25,32-34], and the *maximum supervised posterior *(MSP) learning principle [35,36] has been proposed as discriminative analogue to the MAP learning principle.

In addition to these four learning principles, hybrid learning principles have been proposed to combine the advantages of generative and discriminative learning principles [37-42]. Specifically, the *generative-discriminative trade-off *(GDT) learning principle that interpolates between the ML and the MCL learning principle has been proposed in [37], and the *penalized generative-discriminative trade-off *(PGDT) learning principle that interpolates between the MAP and the MSP learning principle has been proposed in [41].

Here, we introduce a unified generative-discriminative learning principle containing the ML, the MAP, the MCL, the MSP, the GDT, and the PGDT learning principle as limiting cases. We discuss the interpretation of this learning principle, and we investigate its utility using four data sets of TFBSs.

## Results and Discussion

In this section, we present six established learning principles, then introduce the unified generative-discriminative learning principle containing the six established learning principles as special cases, and finally present some discussion and interpretation of the learning principle introduced. We start with considering classifiers that are based on probabilistic models defined by the likelihood *P *(*x* | *c*, *λ*) for sequence *x* given class label *c *and parameter vector *λ*. Based on such models the decision criterion [43] of the classifier is defined as(1)

where *P *(*c *| *x*, *λ*) is the conditional likelihood of class label *c *given sequence *x* and parameter vector *λ*, *P*(*c*, *x* | *λ*) is the likelihood of sequence *x* and class label *c *given parameter vector *λ*, *P *(*c *| *λ*) is the probability of class *c *given parameter vector *λ*, and *P*(*x*|*c*, *λ*) is the conditional probability of sequence *x* given class label *c *and parameter vector *λ*.

The decision and classification performance depend on the parameter vector *λ*. Hence, one needs to infer *appropriate *parameter vectors *λ* from a data set *D*: = (*x*_1_,...,*x*_*N*_) of *N *statistically independent and identically distributed (i.i.d.) sequences and the corresponding class labels *C*:= (*c*_1_,..., *c*_*N*_). In the first subsection, we present six learning principles that have been proposed in the machine-learning community and that are nowadays also used in bioinformatics. In the second subsection, we propose a unified learning principle containing all of these six learning principles as special cases. In the third subsection, we provide a mathematical interpretation of this learning principle, and in the fourth subsection we present four case studies illustrating the utility of this learning principle. We present some implementation details in the **Methods** section.

### Established learning principles

Learning principles can be categorized by two criteria. On the one hand, they can be divided by their objective into generative, discriminative, and hybrid learning principles. Generative learning principles aim at an accurate representation of the distribution of the training data in each of the classes, discriminative learning principles aim at an accurate classification of the training data into the classes, and hybrid learning principles are an interpolation between generative and discriminative learning principles. On the other hand, learning principles can be divided by their utilization of prior knowledge into Bayesian and non Bayesian. We call learning principles that incorporate a prior density *Q *(*λ*|*α*) on the parameter vector *λ* Bayesian, where *α* denotes a vector of hyper parameters, while we call learning principles that only use the data - without any prior - to estimate the parameter vector non Bayesian. In Table 1 we present six established learning principles and their categorization by the above-mentioned criteria, and we describe these learning principles in more detail in the remainder of this subsection.

**Table 1:** Learning principles

		prior knowledge	
		
		non Bayesian	Bayesian
	**generative**	ML	MAP
	
**objective**	**hybrid**	GDT	PGDT
	
	**discriminative**	MCL	MSP

The table shows six established learning principles that can be grouped by their objective as being generative, hybrid, or discriminative and utilization of prior knowledge with the two possibilities non Bayesian and Bayesian. The four elementary learning principles are the generative, non Bayesian maximum likelihood (ML) learning principle, the generative, Bayesian maximum a posteriori (MAP) learning principle, the discriminative, non Bayesian maximum conditional likelihood (MCL) learning principle, and the discriminative, Bayesian maximum supervised posterior (MSP) learning principle. The hybrid learning principles which interpolate between generative and discriminative learning principles are the non Bayesian generative-discriminative trade-off (GDT) learning principle and the penalized generative-discriminative trade-off (PGDT) learning principle.

#### Generative learning principles

The maximum likelihood (ML) learning principle is one of the first learning principles used in bioinformatics. Originally, it was proposed by R. A. Fisher at the beginning of the 20^*th *^century [28,29]. The ML learning principle aims at finding the parameter vector  that maximizes the likelihood of the labeled data set (*C*, *D*) given the parameter vector *λ*,(2)

However, for many applications, the amount of sequence data available for training is very limited. For this reason, the ML learning principle often leads to suboptimal classification performance e.g. due to zero-occurrences of some nucleotides or oligonucleotides in the training data sets.

The maximum a posteriori (MAP) learning principle, which applies a prior *Q *(*λ*|*α*) to the parameter vector, establishes a theoretical foundation to alleviate this problem and at the same time allows the inclusion of prior knowledge aside from the training data [30]. The MAP learning principle aims at finding the parameter vector  that maximizes the posterior density,(3)

If for a given family of likelihood functions *P*(*C*, *D*|*λ*) the posterior *P*(*λ*|*C*, *D*, *α*) is in the same family of distributions as the prior *Q *(*λ*|*α*), i.e., if

(4),

the prior is said to be *conjugate *to this family of likelihood functions, for hyper parameter vector  incorporates both prior knowledge and training data. Conjugate priors often allow an interpretation of the hyper parameter vector as stemming from an a priorily observed set of "*pseudo data." *In addition, it allows finding the optimal parameter vector  analytically provided one can determine the maximum of the prior analytically.

#### Discriminative learning principles

Discriminative learning principles have been shown to be promising in the field of bioinformatics [16,17,20,26,31]. The discriminative analogue to the ML learning principle is the maximum conditional likelihood (MCL) learning principle [24,25,32-34] that aims at finding the parameter vector  that maximizes the conditional likelihood of the labels *C* given the data *D* and parameter vector *λ*,(5)

The effects of limited data may be even more severe when using the MCL learning principle compared to generative learning principles [23]. To overcome this problem, the maximum supervised posterior (MSP) learning principle [35,36] has been proposed as discriminative analogue to the MAP learning principle. In analogy to equation (3), the MSP learning principle aims at finding the parameter vector  that maximizes the product of the conditional likelihood and the prior density,(6)

#### Generative-discriminative trade-offs

Different hybrid learning principles have been proposed in the machine learning community [37,39,41]. Hybrid learning principles aim at combining the strengths of generative and discriminative learning principles. Here, we follow the ideas of Bouchard and co-workers who propose an interpolation between the generative ML learning principle and the discriminative MCL learning principle [37] as well as the generative MAP learning principle and the discriminative MSP learning principle [41]. The generative-discriminative trade-off (GDT) learning principle proposed in [37] aims at finding the parameter vector *λ* that maximizes the weighted product of the conditional likelihood and likelihood, i.e.,

(7),

for given weight *γ *∈ [0, 1]. As special cases of the PGDT learning principle, we obtain the ML learning principle for *γ *= 1 and the MCL learning principle for *γ *= 0. By varying *γ *between 0 and 1, different beneficial   trade-offs can be obtained for classification.

In close analogy to the MAP and the MSP learning principle, which are obtained by multiplying a prior to the likelihood and conditional likelihood, respectively, the penalized generative-discriminative trade-off (PGDT) learning principle aims at finding the parameter vector *λ* that maximizes the objective function(8)

for given weight *γ *∈ [0, 1]. As special cases of the PGDT learning principle, we obtain the MAP learning principle for *γ *= 1 and the MSP learning principle for *γ *= 0.

We summarize the six established learning principles in Table 1.

### Unified generative-discriminative learning principle

Comparing equations (2), (3), (5), (6), (7), and (8), we find that the following three terms are sufficient for defining these six learning principles:

1. the conditional likelihood *P *(*C*|*D*, *λ*),

2. the likelihood *P *(*C*, *D*|*λ*), and

3. the prior *Q *(*λ*|*α*).

With the goal of unifying and generalizing all six learning principles, we propose a unified generative-discriminative learning principle that aims at finding the parameter vector *λ* that maximizes the weighted product of the conditional likelihood, likelihood, and prior, i.e.,

(9),

with the weighting factors *β*:= (*β*_0_, *β*_1_, *β*_2_), *β*_0_, *β*_1_, *β*_2 _∈ , and *β*_0_, + *β*_1_ + *β*_2 _= 1.

The six established learning principles can be obtained as limiting cases of equation (9) as follows

• ML if *β* = (0, 1, 0),

• MAP if *β* = (0, 0.5, 0.5),

• MCL if *β* = (1, 0, 0),

• MSP if *β* = (0.5, 0, 0.5),

• GDT if *β*_2 _= 0, and

• PGDT if *β*_2 _= 0.5.

**Figure 1:**
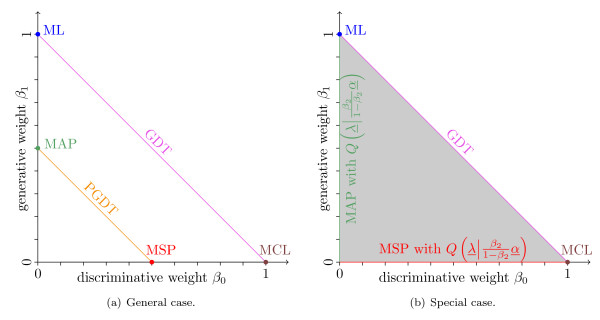
**Illustration of the unified generative-discriminative learning principle**. The plots show a projection of the simplex *β* onto the (*β*_0_, *β*_1_)-plane and the corresponding learning principles for the specific weights encoded by colors. Figure 1(a) shows the general interpretation of the simplex where the points (0, 1), (0, 0.5), (1, 0), and (0.5, 0) refer to the ML, MAP, MCL, and MSP learning principle, respectively, while the lines *β*_1 _= 1 - *β*_0 _and *β*_1 _= 0.5 - *β*_0 _refer to the GDT and PGDT learning principle, respectively. Figure 1(b) shows the interpretation of the unified generative-discriminative learning principle for a conjugate prior that satisfies the condition of equation (11). In this case, each point on the abscissa (*β*_0_-axis) and ordinate (*β*_1_-axis) refers to the MSP and MAP learning principle, respectively, using the prior in a weighted version . The simplex colored in gray corresponds to the MSP learning principle using the weighted posterior  as prior for the parameter vector *λ*.

In Figure 1(a), we illustrate the simplex *β* by a projection onto the (*β*_0_, *β*_1_)-plane showing the established learning principles as well as the unified generative-discriminative learning principle. However, there are several other hybrid learning principles that are not covered by this unification.

### Interpretation of the unified generative-discriminative learning principle

In this subsection, we investigate the simplex *β* and its relation to six established learning principles. First, we consider the axes of the simplex *β*. We can write the learning principle that corresponds to the *β*_0_-axis (*β*_0 _> 0 and *β*_1 _= 0) using the constraint *β*_0 _= 1 - *β*_2 _for this axis as(10a)

Similarly, we can write the learning principle that corresponds to the *β*_1_-axis (with *β*_0 _= 0 and *β*_1 _> 0) as(10b)

These equations state that each point on the abscissa (*β*_0_-axis) and on the ordinate (*β*_1_-axis) corresponds to the MSP and the MAP learning principle, respectively, with a weighted prior.

If the prior fulfills the condition(11)

for any *ξ *∈ ℝ^+^, each point (1 - *β*_2_, 0) and (0, 1 - *β*_2_) on the axes corresponds to either the MSP or the MAP learning principle using the prior , respectively. The *Generalized Dirichlet prior *for Markov random fields [27], which has been proposed to allow a direct comparison of the MAP and the MSP learning principle, fulfills the condition of equation (11) (Appendix A in Additional File 1).

Second, we consider the lines *β*_1 _= *ν *- *β*_0 _with *ν *∈ [0, 1]. As visualized in Figure 1(a), the unified generative-discriminative learning principle results in the GDT and the PGDT learning principle for *ν *= 1 and *ν *= 0.5, respectively. Using *β*_2 _∈ (0, 1) and the condition of equation (11) with , we find that equation (9) can be written as(12)

This equation is equivalent to equation (8), stating that - for each *β*_2 _- each point on the line *β*_1 _= (1 - *β*_2_) - *β*_0 _corresponds to a specific instance of the PGDT learning principle with prior . Using this result, the unified generative-discriminative learning principle allows an in-depth analysis of the PGDT learning principle using different priors.

Finally, we consider a second interpretation of the unified generative-discriminative learning principle. The last two terms of the equation (9) consisting of the weighted likelihood and the weighted prior might be interpreted as a weighted posterior. Using the assumption of conjugacy (equation (4)), the condition of equation (11), and *β*_0_, *β*_1_, *β*_2 _∈ ℝ^+^, we obtain(13a)

stating that each point on the simplex can be interpreted as MSP learning principle with an informative prior  composed of the likelihood and the original prior. Interestingly, the interpretation of each point of the simplex as instance of the MSP learning principle using the weighted posterior as prior remains valid even for priors that do not fulfill the conditions. Figure 1(b) visualizes these results.

### Testing

In this subsection, we present four case studies illustrating the utility of the unified generative-discriminative learning principle. In specific practical applications, the choice of appropriate training and test data sets is a highly non-trivial task. Since the final results strongly depend on the chosen data sets, we recommend this choice to be made with great care and in a problem-specific manner. This choice is typically influenced by a-priori knowledge on both the expected binding sites (BSs) and the targeted genome regions. Examples of features that are often considered when choosing appropriate data sets are the GC content of the target region, their association with CpG islands, or their size and proximity to transcription start sites.

Carefully choosing appropriate training and test data sets is of additional advantage if the set of targeted genome regions is not homogeneous, e.g., comprising both GC-rich and GC-poor regions, CpG islands and CpG deserts, TATA-containing and TATA-less promoters, upstream regions with and without BSs of another TF, etc. In this case, one often finds that different learning principles work well for different subgroups, even if the same combination of models is chosen, providing the possibility of choosing subgroup-specific learning principles by choosing different values of *β*.

These considerations are vital for a successful prediction of TFBSs, but beyond the scope of this paper, so we choose some traditional data sets in the following case study. Specifically, we choose the following four data sets of experimentally verified TFBSs of length *L *= 16 bp from TRANSFAC [44]. The data set AR/GR/PR contains 104 BSs from three specific steroid hormone receptors from the same class of TFs. The data sets GATA and Thyroid contain 110 and 127 BSs, respectively, of TFs with zinc-coordinating DNA-binding domains. Finally, the data set NF-*κ*B contains 72 BSs of the rapid-acting family of primary TFs NF*κ*B. As background data set we choose the standard background data set of TRANSFAC consisting of 267 second exons of human genes with 68,141 bp in total, which we chunk into sequences of length of at most 100 bp. We build classifiers with the goal of classifying, for each family of TFs separately, a given 16-mer as BS or as subsequence of a background sequence.

We choose a naïve Bayes classifier consisting of two PWM models and the Generalized Dirichlet prior [27] using an *equivalent sample size *(ESS) (Appendix A in Additional File 1) of 4 and 1024 for the foreground and the background class, respectively. We choose the sensitivity for a specificity of 99.9% [19] as performance measure. We present the results for three additional performance measures in Appendix B of Additional File 1. We perform a 1,000-fold stratified hold-out sampling with 90% of the data for training and 10% of the data for assessing the performance measures for the evaluation of the unified generative-discriminative learning principle.

**Figure 2:**
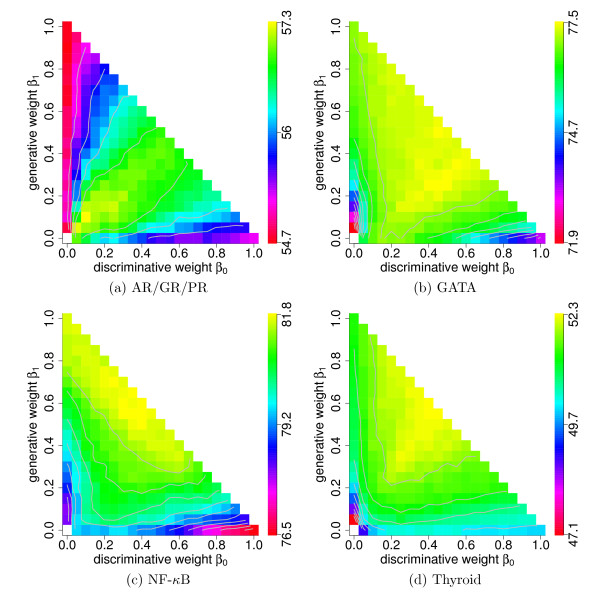
**Performance of the unified generative-discriminative learning principle for four data sets**. We perform a 1,000-fold stratified hold-out sampling procedure for the four data sets, record for different values of *β* the mean sensitivity for a fixed specificity of 99.9%, and plot the mean sensitivities on the simplex *β* in analogy to Figure 1. Yellow indicates the highest sensitivity, red indicates the lowest sensitivity, and the gray contour lines of each subfigure indicate multiples of the standard error of the maximum sensitivity.

In Figure 2a, we illustrate the results for BSs of the TFs AR/GR/PR. Considering the ML learning principle located at (*β*_0_, *β*_1_) = (0, 1) and the MCL learning principle located at (*β*_0_, *β*_1_) = (1, 0), we find a sensitivity of 54.7% and 55.2%, respectively. Interestingly, the MCL learning principle achieves a higher sensitivity for a given specificity of 99.9% than the ML learning principle for this small data set. Using the Generalized Dirichlet prior with hyper parameters corresponding to uniform pseudo data, the sensitivities can be increased. Considering the MAP learning principle located at (*β*_0_, *β*_1_) = (0, 0.5) and the MSP learning principle located at (*β*_0_, *β*_1_) = (0.5, 0), we obtain a sensitivity of 54.9% and 55.6%, respectively. This shows that the MSP learning principle yields an increase of sensitivity of 0.7% compared to the MAP learning principle, consistent with the general observation that discriminatively learned classifiers often outperform their generatively learned counterparts. This increase of sensitivity is achieved using the same prior and the same hyper parameters for both learning principles, but it is possible that the particular choice of the hyper parameters may favour one of the learning principles.

Following equations (10a) and (10b), each point on the *β*_0_- and *β*_1_-axis corresponds to the MSP and the MAP learning principle, respectively, with specific hyper parameters *α*. The location on the axis indicates the strength of the prior reflected by the *virtual *ESS (Appendix A in Additional File 1). Next, we investigate for both learning principles the influence of the strength of the prior on the sensitivity.

For the MAP learning principle, the sensitivity ranges from 54.7% for *β* = (0, 0.05, 0.95) to 54.8% for *β* = (0, 0.95, 0.05), achieving a maximum of 55.1% for *β* = (0, 0.1, 0.9). For the MSP learning principle, the sensitivity ranges from the maximum value 56.7% for *β* = (0.05, 0, 0.95) to 55.3% for *β* = (0.95, 0, 0.05). Comparing the maximum sensitivities for both learning principles and different virtual ESSs, we find that the MSP learning principle with a maximum sensitivity of 56.7% clearly outperforms the MAP learning principle by 1.6%, whereas the difference of sensitivities is only 0.7% for the original ESS.

Investigating this increase in the difference of sensitivities between the results for the MAP and the MSP learning principle, we find that the sensitivity increases for decreasing *β*_0 _on the *β*_0_-axis, which corresponds to the MSP learning principle with an increasing virtual ESS of the prior. In contrast to this observation, the sensitivity for the MAP learning principle increases less strongly with an increasing virtual ESS. This finding gives a first hint that a prior with a large ESS might be beneficial for the MSP learning principle, while we cannot observe a similar effect for the MAP learning principle in this case.

Next, we consider the lines *β*_1 _= *ν *- *β*_0_, which correspond to the hybrid learning principles GDT and PGDT for *ν *= 1 and *ν *= 0.5, respectively. For the GDT learning principle, the sensitivity ranges from 54.7% for *β* = (0, 1, 0) to 55.2% for *β* = (1, 0, 0), reaching a maximum of 56.9% for *β* = (0.55, 0.45, 0). For the PGDT learning principle, the sensitivity ranges from 54.9% for *β* = (0, 0.5, 0.5) to 55.6% for *β* = (0.5, 0, 0.5), reaching a maximum of 57.1% for *β* = (0.3, 0.2, 0.5). For both learning principles, we find that the sensitivity is initially increasing and finally decreasing. This observation indicates that neither the MAP nor the MSP learning principle with a Generalized Dirichlet prior representing uniform pseudo data is optimal for estimating the parameter vector *λ*.

Next, we investigate the interior of the simplex. We vary both *β*_0 _and *β*_1 _along a grid with step width 0.05, and we find the highest sensitivity of 57.3% for *β* = (0.1, 0.1, 0.8). We find the region of highest sensitivity clearly inside the simplex near the angle bisector. This region corresponds to the MSP learning principle with an informative prior based on weighted likelihood and weighted original prior. Comparing the highest sensitivity for the GDT, the PGDT, and the unified generative-discriminative learning principle, we find that it increases from 56.9% over 57.1% to 57.3%, confirming that the prior can have a positive influence on the performance.

Turning to the results of the other three TFs GATA, NF-*κ*B, and Thyroid, we find qualitatively similar results. The highest sensitivities are located inside the simplex, while the lowest sensitivities are located on the axes. For BSs of the TF GATA, we obtain a sensitivity of 77.5% for *β* = (0.45, 0.25, 0.3), for the BSs of the TF NF-*κ*B, we obtain a sensitivity of 81.8% for *β* = (0.4, 0.55, 0.05), and for the BSs of the TF Thyroid, we obtain 52.3% for *β* = (0.4, 0.55, 0.05). Similar to the data set of AR/GR/PR, we find a small region with high sensitivity for the BSs of the TFs NF-*κ*B and Thyroid, while we find a broad region with high sensitivity for the BSs of the TF GATA.

We summarize the sensitivities for the ML, the MCL, the MAP, the MSP, and the unified generative-discriminative learning principle in Table 2. We find that for all four TFs the unified generative-discriminative learning principle yields the highest sensitivities. Regarding the *β*_1_-axis, which corresponds to the MAP learning principle using the Generalized Dirichlet prior representing uniform pseudo data with different ESSs, we find that increasing the prior weight *β*_2_, which is equivalent to decreasing the generative weight *β*_1_, often reduces the sensitivity. We obtain the lowest sensitivity for the MAP learning principle for the largest prior weights *β*_2 _in almost all cases. In contrast to this observation, we find on the *β*_0_-axis, which correspond to the MSP learning principle with the Generalized Dirichlet prior representing uniform pseudo data with different ESSs, that increasing the prior weight *β*_2 _improves the sensitivity at least initially.

**Table 2:** Results for four data sets

	AR/GR/PR	GATA	NF-*κ*B	Thyroid
ML	54.7	77.0	81.6	51.3

MCL	55.2	73.2	76.5	50.0

MAP	55.1	77.0	81.6	51.3

MSP	56.9	77.0	79.6	50.4

Unified	**57.3**	**77.5**	**81.8**	**52.3**

Summary the results of Figure 2 for the 4 data sets containing the highest sensitivity for the ML, the MCL, the MAP, the MSP, and the unified generative-discriminative learning principle. For the MAP, the MSP, and the unified generative-discriminative learning principle, we present the best results form the simplex *β* which correspond to one of these learning principles (see Figure 1b). For each data set, the highest sensitivity is displayed in bold.

Interestingly, we obtain qualitatively similar results when using other performance measures (Appendix B in Additional File 1). These observations suggest that the same classifier trained either by generative or by discriminative learning principles may prefer different ESSs even if one uses a prior that corresponds to uniform pseudo data. Hence, the strength of the prior has a decisive influence on comparisons of the results from generative and discriminative learning principles as well as the results of Bayesian hybrid learning principles as for instance PGDT learning principle. Most importantly, we find that the unified generative-discriminative learning principle leads to an improvement for almost all of the studied data sets and performance measures.

## Conclusions

A plethora of algorithms for the recognition of short DNA sequence motifs has been proposed in the last decades. These algorithms differ by their underlying statistical models and the employed learning principles. In bioinformatics, generative learning principles have a long tradition, but recently it was shown that discriminative learning principles can lead to an improvement of the recognition of short signal sequences.

We introduce a unified generative-discriminative learning principle that contains the ML, the MAP, the MCL, the MSP, the GDT, and the PGDT learning principle as limiting cases. This learning principle interpolates between the likelihood, the conditional likelihood, and the prior, spanning a three-dimensional simplex, which allows a more detailed comparison of different learning principles. Furthermore, we find that under mild assumptions each point on the simplex can be interpreted as MSP learning principle using an informative prior composed of a weighted likelihood and a weighted original prior.

We find that the unified generative-discriminative learning principle improves the performance of classifiers for the recognition of vertebrate TFBSs over any of the six established learning principles it contains as special case. We make all implementations available for the scientific community as part of the open-source Java library Jstacs [45], which allows using this learning principle easily for other bioinformatics problems. Although we demonstrate the utility of the unified generative-discriminative learning principle only for four data sets of TFBSs and four performance measures, it is conceivable that it can be successfully applied to other multinomial data such as data of transcription start sites, donor and acceptor splice sites, splicing enhancers and silencers, as well as binding sites of insulators, nucleosomes, and miRNAs, as well as continuous data.

## Methods

Considering the task of determining the optimal parameter vector , we find that generative learning principles often allow to estimate  analytically for simple models such as Markov models, but one must use numerical optimization procedures for discriminative and hybrid learning principles, and consequently for the unified generative-discriminative learning principle as well. If the conditional likelihood, the likelihood, and the prior are log-convex functions, we can use any numerical algorithm to determine the globally optimal parameter vector  for the unified generative-discriminative learning principle.

Different numerical methods including steepest descent, conjugate gradient, quasi-Newton methods, and limited-memory quasi-Newton methods have been evaluated in [46]. In the case studies presented in the previous subsection, we use a limited-memory quasi-Newton method. In analogy to [37], we fix *β* for the unified generative-discriminative learning principle, and we compute the results for a grid of given values of *β*, providing an overall impression of the performance for the whole simplex *β*.

The unified generative-learning principle can in principle be used for all types of data, and it is not limited to multinomial data presented in section *Testing*. We make all implementations available for the scientific community as part of the open-source Java library Jstacs [45]. Jstacs comprises an efficient representation of sequence data and provides object-oriented implementations of many statistical models. We implement the unified generative-discriminative learning principle as a multi-threaded class based on the Jstacs class hierarchy [47]. This allows applying the learning principle efficiently on multi-core computers and to other statistical models. For optimizing parameters, we use optimization procedures provided by Jstacs.

## List of abbreviations used

BS: binding site; ESS: equivalent sample size; GDT: generative-discriminative trade-off; MAP: maximum a posteriori; MCL: maximum conditional likelihood; ML:  maximum likelihood; MSP: maximum supervised posterior; PGDT: penalized generative-discriminative trade-off; PWM: position weight matrix; TF: transcription factor; TFBS: transcription factor binding sites; WAM: weight array matrix.

## Availability and Requirements

Project name: GenDisMix

Project home page: [47]

Operating system(s): Platform independent

Programming language: Java 1.5

Requirements: Jstacs 1.3

License: GNU General Public License version 3

## Authors' contributions

JK and IG developed the basic ideas. JK and JG implemented the software. JK performed the case studies. All authors contributed to data analysis, writing, and approved the final manuscript.

## Supplementary Material

Additional file 1 **Appendix**. This file contains additional information about Markov random fields and the case studies. Click here for file
