# Supplementary material for: Unifying generative and discriminative learning principles
Source: BMC Bioinformatics. 2010 Feb 22;11:98. doi: 10.1186/1471-2105-11-98 (PMC2848239; doi:10.1186/1471-2105-11-98)
Supplement: Additional file 1 — Appendix. This file contains additional information about Markov random fields and the case studies. [file 1471-2105-11-98-S1.PDF]

Appendix A: Markov random fields and prior used in the case study

Many of the models currently used in bioinformatics such as position weight matrix (PWM) models [1, 2, 3], weight array matrix (WAM) models [4, 5, 6], inhomogeneous and permuted Markov models of higher order [7, 8], Bayesian trees [9], their variable order extensions [10], and maximum entropy models [11] are special cases of Markov random fields (MRFs).

MRFs belong to the family of graphical models, where random variables are represented by nodes of a graph, and the dependency structure of the joint probability distribution is represented by edges [12]. MRFs are undirected graphical models, i.e., the underlying graph is undirected, where edges between nodes model potential conditional statistical dependencies between the random variables represented by these nodes, while the absence of edges between nodes represents conditional independences of the associated random variables given their neighboring nodes. The undirected graph of an MRF is determined by indicator functions $\underline{f} := (\underline{f}_1, \ldots, \underline{f}_{|\mathcal{C}|})$, where $\underline{f}_c := (f_{c,1}, \ldots, f_{c,|\underline{f}_c|})$ denote the indicator functions of the class c. Following the notation of [13, 14], an indicator function $f_{c,i}(\underline{x})$ determines whether the parameter $\lambda_{c,i}$ is used for sequence $\underline{x} \in M$, and the likelihood of an MRF determined by the indicator functions $\underline{f}$ is given by

$$P\left(c, \underline{x} \middle| \underline{\lambda}\right) = \frac{\exp\left(\lambda_c + \sum_{i=1}^{|\underline{f}_c|} \lambda_{c,i} \cdot f_{c,i}(\underline{x})\right)}{Z(\underline{\lambda})}. \tag{1a}$$

Using this parameterization, the log-likelihood of a labeled data set

$$\log P\left(\underline{C}, \underline{D} \middle| \underline{\lambda}\right) = \sum_{n=1}^{N} \log P\left(c_n, \underline{x}_n \middle| \underline{\lambda}\right) \tag{1b}$$

as well as the log-conditional likelihood

$$\log P\left(\underline{C} \middle| \underline{D}, \underline{\lambda}\right) = \sum_{n=1}^{N} \log P\left(c_n \middle| \underline{x}_n, \underline{\lambda}\right) = \sum_{n=1}^{N} \log \left(\frac{P\left(c_n, \underline{x}_n \middle| \underline{\lambda}\right)}{\sum_c P\left(c, \underline{x}_n \middle| \underline{\lambda}\right)} \right) \tag{1c}$$

are convex functions.

Using an MRF with a discrete codomain M and the MAP, the MSP, or the unified generative-discriminative learning principle, we choose the Generalized Dirichlet prior [15]

$$Q\left(\underline{\lambda} \middle| \underline{\alpha}\right) \propto Z(\underline{\lambda})^{-\alpha_\cdot} \cdot \exp\left(\sum_{c \in \mathcal{C}} \alpha_c \lambda_c + \sum_{i=1}^{|\underline{f}_c|} \alpha_{c,i} \lambda_{c,i} \right), \tag{2}$$

as prior for the parameter vector $\underline{\lambda}$ of an MRF. For the hyper-parameter $\underline{\alpha}$, we follow the description of [15] and choose the hyper-parameters according to the BDeu prior [16, 17]. This choice states that each class c has a class-specific *equivalent sample size* (ESS) α_c uniformly distributed among all possible sequence from the codomain M determining the values of all remaining hyper-parameter $\alpha_{c,i}$. The ESS $\alpha_\cdot$ of the complete prior is

the sum of all class-specific ESSs, $\alpha_{.} := \sum_{c \in \mathcal{C}} \alpha_c$. This prior fulfills the condition of equation 11, which allows to interpret the unified generative-discriminative learning principle using this prior for MRFs as illustrated in Figure 1(b). For the Generalized Dirichlet prior, we can interpret the condition of equation 11 as multiplication of the initially chosen ESS by a factor resulting in a *virtual* ESS. This allows to interpret the learning principle corresponding to each point of the axes as instances of the MSP or MAP learning principle, respectively, using different ESSs but the same ratio between the hyper-parameters. In the interpretation of each point of the simplex $\underline{\beta}$ (equation 13a and 13b), the hyper-parameters $\underline{\tilde{\alpha}}$ are

$$\tilde{\alpha}_c := N_c + \frac{\beta_2}{\beta_1}\alpha_c \quad \text{and} \quad \tilde{\alpha}_{c,i} := N_{c,i} + \frac{\beta_2}{\beta_1}\alpha_{c,i} \tag{3a}$$

with

$$N_c := \sum_{n=1}^{N} \delta_{c_n,c} \quad \text{and} \quad N_{c,i} := \sum_{n=1}^{N} \delta_{c_n,c} f_{c_n,i}(\underline{x}_n), \tag{3b}$$

where δ denotes the Kronecker symbol which is equal to 1 if both indices are equal and otherwise 0

In case of DNA sequences, the codomain M is Σ^L, where $\Sigma = \{A, C, G, T\}$ denotes the alphabet and L denotes sequence length. The case studies presented in this paper are restricted to PWM models defined by the indicator functions $f_{c,i}(\underline{x}) := \delta_{x_\ell,b}$, for all position $\ell \in [1, L]$ and all symbols $b \in \Sigma$.

Appendix B: Different performance measures for the case study

Here, we present the results of the 1,000-fold stratified hold-out sampling for 4 TFs and for 4 performance measures, the sensitivity (Sn) for a specificity of 99.9%, the false positive rate (fpr) for a fixed Sn of 95%, the positive predictive value (ppv) for a fixed Sn of 95%, and the area under the precison-recall curve (auc-PR) [15, 18].

Figure S1 shows the results of the stratified hold-out sampling, where each column contains the subfigures for one data set corresponding to one transcription factor, and each row contains the subfigures for a specific performance measure. In each subfigure, yellow indicates the best results, red indicates the worst results, and the gray contour lines of each subfigure represent multiples of the standard error of the best result.

In Table S1, we summarize the best results of each subfigure of Figure S1. In 13 out of 16 cases, the best results are achieved for some weighting vector $\underline{\beta}$ in the interior of the simplex. The only cases for which we obtain the best result on one of the axes is for the data set GATA using the performance measure auc-PR and Thyroid using the performance measures fpr and ppv. In none of the cases the best result is obtained for a generative learning principle. Comparing the results of the 16 individual studies to each other, we find that the weighting vector $\underline{\beta}$ that gives the best results depends on the data set as well as the performance measure.

Considering the differences between the best results of the simplex and the best results of the β_0- and β_1-axis, we find that this difference is greater than the standard error of the best result of the simplex for the auc-PR of

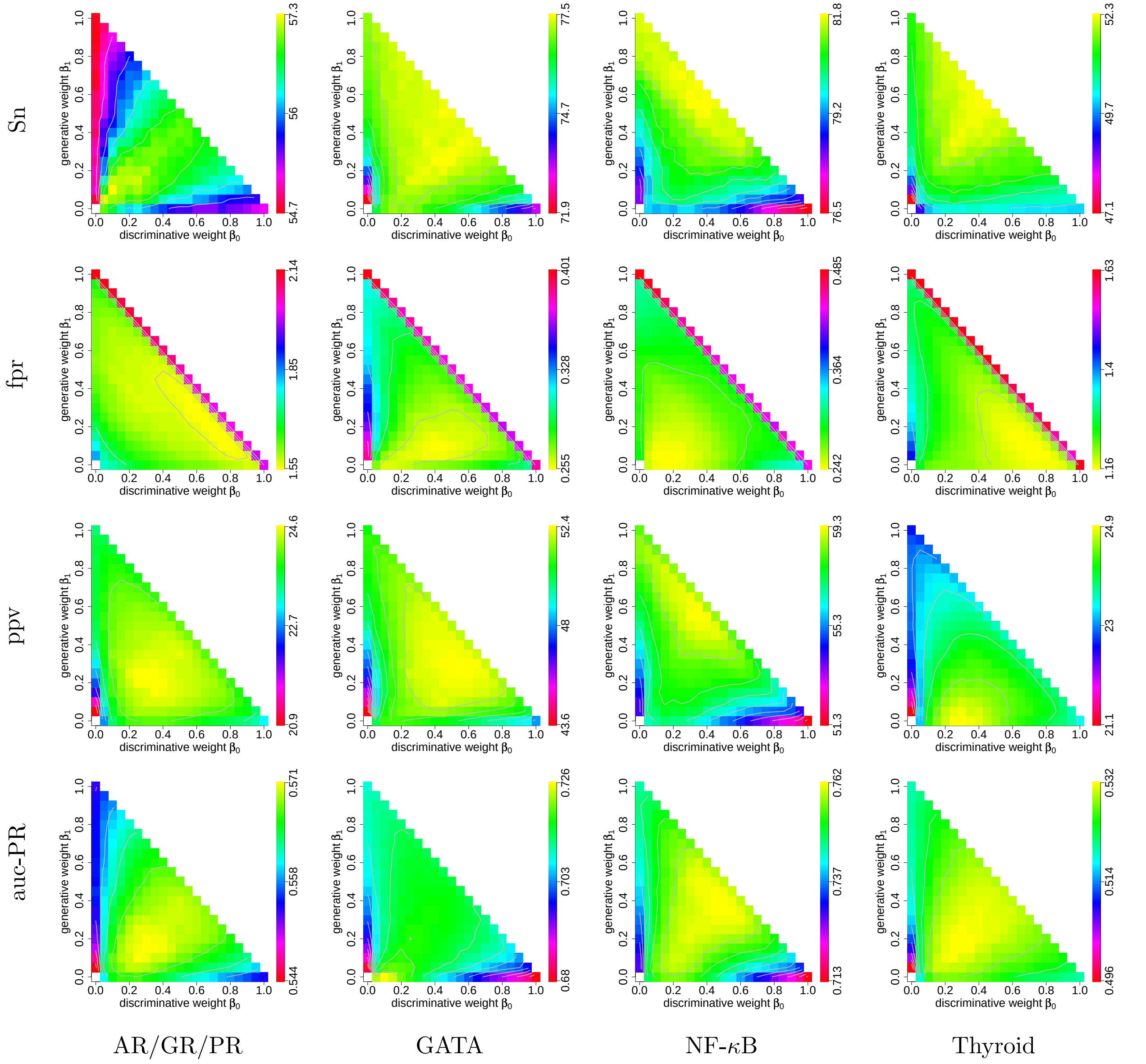

Figure S1: Results of the 1,000-fold stratified hold-out sampling procedure. Each column contains the subfigures for one data sets corresponding to one transcription factor, and each row contains the subfigures for a specific performance measure. In each subfigure, we plot the values of the specific performance measure as a function of $\underline{\beta}$ in analogy to Figure 2. Yellow indicates the best results, red indicates the worst results, and the gray contour lines of a subfigure indicate multiples of the standard error of the best result. We find the best results in the interior of the simplex $\underline{\beta}$ in 13 out of 16 cases.

		AR/GR/PR	GATA	NF-κB	Thyroid
Sensitivity for fixed specificity in %	ML	54.7	77.0	81.6	51.3
	MCL	55.2	73.2	76.5	50.0
	MAP	55.1	77.0	81.6	51.3
	MSP	56.9	77.0	79.6	50.3
	Unified	**57.3**	**77.5**	**81.8**	**52.3**
False positive rate for fixed sensitivity in %	ML	2.14	0.401	0.485	1.63
	MCL	2.01	0.384	0.437	1.63
	MAP	1.61	0.309	0.285	1.28
	MSP	1.57	0.260	0.243	**1.16**
	Unified	**1.55**	**0.255**	**0.242**	**1.16**
Positive predictive value for fixed sensitivity in %	ML	23.5	50.6	58.2	22.6
	MCL	23.1	48.1	51.3	23.4
	MAP	23.7	50.95	58.6	23.0
	MSP	24.1	51.1	57.1	**24.9**
	Unified	**24.6**	**52.4**	**59.3**	**24.9**
Area under PR curve	ML	0.554	0.709	0.746	0.520
	MCL	0.554	0.680	0.713	0.520
	MAP	0.555	0.711	0.747	0.520
	MSP	0.567	**0.727**	0.756	0.528
	Unified	**0.571**	**0.727**	**0.762**	**0.532**

Table S1: Summary of the results of Figure S1. For each of the four data sets and each of the four performance measures, we present the results for the ML, the MCL, the MAP, the MSP, and the unified generative-discriminative learning principle. For the MAP, the MSP, and the unified generative-discriminative learning principle, we present the best results from each of the 16 simplices (Figure 1(b)). We find that the best results, displayed in bold face, are obtained by the unified generative-discriminative learning principle. Results that are at least one standard error greater the corresponding results of the other learning principles are highlighted by gray cells.

the AR-GR-PR data set, the Sn and the ppv of the GATA data set, the auc-PR of the NF-κB data set, and the Sn of the Thyroid data set. This indicates that, at least for the studied data sets and the chosen models, the unified generative-discriminative learning principle is useful for estimating the model parameters. We find in all four data sets that the fpr for the GDT learning principle is significantly worse than for the best weighting factor $\underline{\beta}$, whereas the GDT learning principle performs comparable to other learning principles for the performance measures auc-PR, Sn, and ppv.

References

[1] Stormo G, Schneider T, Gold L, Ehrenfeucht A: **Use of the 'perceptron' algorithm to distinguish translational initiation sites**. *NAR* 1982, **10**:2997–3010, [[http://nar.oxfordjournals.org/cgi/content/abstract/10/9/2997]].

[2] Staden R: **Computer methods to locate signals in nucleic acid sequences**. *NAR* 1984, **12**:505–519, [[http://nar.oxfordjournals.org/cgi/content/abstract/12/1Part2/505?ck=nck]].

[3] Kel AE, Gössling E, Reuter I, Cheremushkin E, Kel-Margoulis OV, Wingender E: **MATCH: A tool for searching transcription factor binding sites in DNA sequences.** *Nucleic Acids Res* 2003, **31**(13):3576–3579.

[4] Zhang M, Marr T: **A weight array method for splicing signal analysis**. *Comput. Appl. Biosci.* 1993, **9**(5):499–509.

[5] Salzberg SL: **A method for identifying splice sites and translational start sites in eukaryotic mRNA**. *Comput. Appl. Biosci.* 1997, **13**(4):365–376, [[http://bioinformatics.oxfordjournals.org/cgi/content/abstract/13/4/365]].

[6] Segal E, Fondufe-Mittendorf Y, Chen L, Thåström A, Field Y, Moore IK, Wang JPZ, Widom J: **A genomic code for nucleosome positioning**. *Nature* 2006, **442**(7104):772–778, [[http://dx.doi.org/10.1038%2Fnature04979]].

[7] Yakhnenko O, Silvescu A, Honavar V: **Discriminatively Trained Markov Model for Sequence Classification**. In *ICDM '05: Proceedings of the Fifth IEEE International Conference on Data Mining*, Washington, DC, USA: IEEE Computer Society 2005:498–505.

[8] Zhao X, Huang H, Speed TP: **Finding Short DNA Motifs Using Permuted Markov Models**. *Journal of Computational Biology* 2005, **12**(6):894–906, [[http://www.liebertonline.com/doi/abs/10.1089/cmb.2005.12.894]]. [PMID: 16108724].

[9] Cai D, Delcher A, Kao B, Kasif S: **Modeling splice sites with Bayes networks** . *Bioinformatics* 2000, **16**(2):152–158, [[http://bioinformatics.oxfordjournals.org/cgi/content/abstract/16/2/152]].

[10] Ben-Gal I, Shani A, Gohr A, Grau J, Arviv S, Shmilovici A, Posch S, Grosse I: **Identification of transcription factor binding sites with variable-order Bayesian networks**. *Bioinformatics* 2005, **21**(11):2657–2666, [[http://bioinformatics.oxfordjournals.org/cgi/content/abstract/21/11/2657]].

[11] Yeo G, Burge CB: **Maximum Entropy Modeling of Short Sequence Motifs with Applications to RNA Splicing Signals**. *Journal of Computational Biology* 2004, **11**(2-3):377–394, [[http://www.liebertonline.com/doi/abs/10.1089/1066527041410418]]. [PMID: 15285897].

[12] Jordan MI: **Graphical Models**. *Statistical Science (Special Issue on Bayesian Statistics)* 2004, **19**:140–155.

[13] Berger A, Della Pietra S, Della Pietra V: **A Maximum Entropy Approach to Natural Language Processing**. *Computational Linguistics* 1996, **22**:39–71, [[http://www.cs.cmu.edu/ aberger/maxent.html]].

[14] Klein D, Manning C: **Maxent Models, Conditional Estimation, and Optimization**. *HLT-NAACL 2003 Tutorial* 2003, [[http://www.cs.berkeley.edu/ klein/papers/maxent-tutorial-slides.pdf]].

[15] Keilwagen J, Grau J, Posch S, Grosse I: **Apples and oranges: avoiding different priors in Bayesian DNA sequence analysis**. *BMC Bioinformatics* 2009, **Submitted**.

[16] Buntine WL: **Theory Refinement of Bayesian Networks**. In *Uncertainty in Artificial Intelligence*, Morgan Kaufmann 1991:52–62, [[citeseer.ist.psu.edu/buntine91theory.html]].

[17] Heckerman D, Geiger D, Chickering DM: **Learning Bayesian Networks: The Combination of Knowledge and Statistical Data**. Tech. rep., Microsoft Research, Advanced Technology Division, Redmond, WA 98052 1995, [[http://www.springerlink.com/content/g4780rju5922q051/]].

[18] Davis J, Goadrich M: **The relationship between Precision-Recall and ROC curves**. In *ICML '06: Proceedings of the 23rd international conference on Machine learning*, New York, NY, USA: ACM 2006:233–240, [[http://dx.doi.org/10.1145/1143844.1143874]].
